# The effect of vortioxetine on overall patient functioning in patients with major depressive disorder

**DOI:** 10.1002/brb3.622

**Published:** 2017-02-02

**Authors:** Ioana Florea, Henrik Loft, Natalya Danchenko, Benoît Rive, Melanie Brignone, Elizabeth Merikle, Paula L. Jacobsen, David V. Sheehan

**Affiliations:** ^1^ H. Lundbeck A/SCopenhagenDenmark; ^2^ Lundbeck SASIssy‐les‐MoulineauxFrance; ^3^ Takeda Development Center AmericasDeerfieldILUSA; ^4^ University of South Florida College of MedicineTampaFLUSA

**Keywords:** functional impairment, major depressive disorder, quality of life, Sheehan Disability Scale, vortioxetine

## Abstract

**Background:**

The objectives of this meta‐analysis of data from randomized, placebo‐controlled studies were to assess the effect of vortioxetine on overall functioning (primary) and functional remission (secondary) using the Sheehan Disability Scale (SDS) in adults with major depressive disorder (MDD).

**Methods:**

Data from nine short‐term (6/8 weeks) pivotal studies that included patient functioning assessments were included in this random‐effects meta‐analysis, which used aggregated study‐level data for all therapeutic vortioxetine doses and a mixed‐effect model for repeated measures using the full analysis set.

**Results:**

A total of 4,216 patients received ≥1 dose of study treatment (1,522 placebo, 2,694 vortioxetine 5–20 mg/day). At study end, the meta‐analysis showed improvement for vortioxetine versus placebo (*n* = 911) in SDS total score (vortioxetine 5 mg, *n *=* *564, change from baseline versus placebo [Δ] −0.24, *p *= NS; 10 mg, *n *=* *445, Δ −1.68, *p *≤* *.001; 15 mg, *n *=* *204, Δ −0.91, *p* = NS; 20 mg, *n *=* *340, Δ −1.94, *p *≤* *.01). Functional remission (SDS total score ≤6) was observed with vortioxetine 10 mg (*n *=* *170/573; odds ratio [OR] relative to placebo 1.7, *p *<* *.001) and 20 mg (*n *=* *144/447; OR 1.6, *p *<* *.05), but not 5 mg (*n *=* *207/757; OR 1.1, *p* = NS) or 15 mg (*n *=* *92/295; OR 1.3, *p* = NS).

**Conclusion:**

Vortioxetine 5–20 mg for 6/8 weeks improved overall patient functioning in patients with MDD. Relative to placebo, vortioxetine 10 and 20 mg demonstrated significant improvement in SDS total score and functional remission.

## 1 Introduction

Patients with major depressive disorder (MDD) experience functional impairment that can negatively affect many aspects of daily life, including school, work, interpersonal relationships, and overall social functioning (Greer, Kurian, & Trivedi, 2010; Kessler et al., 2003; Moussavi et al., 2007). MDD is associated with significant disability and economic burden, which can be attributed in part to functional impairment. In 2010, MDD was ranked as the 11th leading cause of disability‐adjusted life years worldwide (Ferrari et al., 2013), with an associated cost estimated at $210.5 billion in the United States alone, with approximately half of the cost attributable to disability in the workplace (Greenberg et al., 2015). Restoration of patient functioning is a desirable goal of MDD treatment (Lam, Filteau, & Milev, 2011); however, the impact of antidepressant therapy on this outcome remains poorly understood. Although improvement in depressive symptoms would be expected to result in improved patient functioning, published evidence suggests that this relationship is variable and complex (Guico‐Pabia, Fayyad, & Soares, 2012; Judd et al., 2008; McKnight & Kashdan, 2009; Romera et al., 2010; Sheehan et al., 2011; Trivedi et al., 2009; Zimmerman et al., 2006, 2008, 2012). Notably, functional impairment may persist beyond the resolution of depressive symptoms in many patients (Judd et al., 2008; McKnight & Kashdan, 2009; Romera et al., 2010; Sheehan et al., 2011).

In clinical studies, the assessment of the beneficial effects of antidepressant therapy on functional impairment in patients with MDD is becoming increasingly common. Among the myriad of quality of life, social functioning, and occupational functioning scales available (Lam et al., 2011), the Sheehan Disability Scale (SDS; Sheehan, Harnett‐Sheehan, & Raj, 1996) has emerged as a useful tool for this purpose. The SDS is a patient‐rated measure of functional disability that assesses the individual's impairment with regard to their work and/or school activities, social life and leisure activities, and functioning in family life and home responsibilities (Sheehan & Sheehan, 2008; Sheehan et al., 1996). The SDS is validated for use in patients with MDD and is sensitive to the effects of antidepressant treatment (Sheehan & Sheehan, 2008).

The multimodal antidepressant vortioxetine is approved for the treatment of adults with MDD in the United States and for the treatment of major depressive episodes in adults in the European Union. It inhibits the serotonin (5‐HT) transporter, is an antagonist at 5‐HT_3_, 5‐HT_7_, and 5‐HT_1D_ receptors, is a partial agonist at 5‐HT_1B_ receptors, and is an agonist at 5‐HT_1A_ receptors (Bang‐Andersen et al., 2011; Mork et al., 2012; Westrich et al., 2012). In preclinical studies, vortioxetine has demonstrated effects on serotonergic, noradrenergic, dopaminergic, cholinergic, norepinephrinergic, acetylcholinergic, histaminergic, gabanergic, and glutamatergic neurotransmission (Bang‐Andersen et al., 2011; Mork et al., 2012; Sanchez, Asin, & Artigas, 2015). The efficacy and safety of vortioxetine in patients with MDD has been demonstrated in a series of short‐ and long‐term studies (Alvarez et al., 2012; Boulenger, Loft, & Olsen, 2014; Henigsberg et al., 2012; Jacobsen et al., 2015; Katona, Hansen, & Olsen, 2012; Mahableshwarkar, Jacobsen, Chen, et al., 2015; McIntyre, Lophaven, & Olsen, 2014; Montgomery, Nielsen, et al., 2014). The effects of vortioxetine on general functioning were assessed (with the SDS) as secondary measures in a subset of short‐term clinical trials (Baldwin, Loft, & Dragheim, 2012; Boulenger et al., 2014; Henigsberg et al., 2012; Jacobsen et al., 2015; Jain et al., 2013; Mahableshwarkar, Jacobsen, & Chen, 2013; Mahableshwarkar, Jacobsen, Chen, et al., 2015; Mahableshwarkar, Jacobsen, Serenko, et al., 2015; Takeda, 2013). The purpose of this post hoc meta‐analysis was to analyze the SDS results from these studies to determine the effect of vortioxetine on overall patient functioning as well as the proportion of patients who achieved functional remission versus placebo.

## 2 Materials and Methods

### 2.1 Datasets

The clinical development program for vortioxetine in MDD was conducted in multiple countries. The studies were designed and carried out in accordance with the principles of the World Medical Association *Declaration of Helsinki*, the principles of International Conference on Harmonisation Harmonised Tripartite Guideline for *Good Clinical Practice*, and all applicable local or regional regulatory requirements. The study sponsors (Takeda Pharmaceutical and H. Lundbeck A/S) had overall responsibility for the studies, including those where monitoring was delegated to a contract research organization. The protocols, statistical analyses, and statistical reporting for all studies were developed in accordance with current scientific research approaches and relevant guidelines.

All pivotal vortioxetine studies that assessed the SDS were included in this meta‐analysis. Thus, data from nine short‐term (6/8 weeks) multicenter, double‐blind, randomized, placebo‐controlled, parallel‐group, fixed‐dose clinical studies of vortioxetine conducted in adults (aged 18–75 years) with MDD were analyzed. The details of the study design and the results of the nine included trials have been previously published (Baldwin et al., 2012; Boulenger et al., 2014; Henigsberg et al., 2012; Jacobsen et al., 2015; Jain et al., 2013; Mahableshwarkar, Jacobsen, Chen, et al., 2015; Mahableshwarkar, Jacobsen, Serenko, et al., 2015; Mahableshwarkar et al., 2013; Takeda, 2013). Table 1 provides a summary of treatment dosages, number of participants in each dosage arm, treatment duration, and key inclusion criteria for each of the trials included in this meta‐analysis. All patients enrolled in these studies met the *Diagnostic and Statistical Manual of Mental Disorders, Fourth Edition, Text Revision* (DSM‐IV‐TR; American Psychiatric Association (APA), 2000) criteria for a major depressive episode lasting ≥3 months and were ≥18 years old. Additionally, patients were required to have Montgomery‐Åsberg Depression Rating Scale (MADRS; Montgomery & Asberg, 1979) scores ≥22 (NCT00672620, Mahableshwarkar et al., 2013), ≥30 (NCT00672958, Jain et al., 2013) or ≥26 (all other trials; Baldwin et al., 2012; Boulenger et al., 2014; Henigsberg et al., 2012; Jacobsen et al., 2015; Mahableshwarkar, Jacobsen, Chen, et al., 2015; Mahableshwarkar, Jacobsen, Serenko, et al., 2015; Takeda, 2013; Table 1).

**Table 1:** Summary characteristics of the nine short‐term, placebo‐controlled studies of vortioxetine in patients with MDD included in the meta‐analysis (APTS)

NCT identifier	Treatment period	Dose mg/day (*n*)	Key inclusion criteria for MDD	Reference
NCT00635219	8 weeks	VOR 2.5 (155)VOR 5 (157)VOR 10 (151)DUL 60 (155)PBO (148)	MADRS ≥26MDE ≥3 months	Baldwin et al. (2012)
NCT00735709	8 weeks	VOR 1 (140)VOR 5 (140)VOR 10 (139)PBO (140)	MADRS ≥26MDE ≥3 months	Henigsberg et al. (2012)
NCT01140906	8 weeks	VOR 15 (151)VOR 20 (151)DUL 60 (147)PBO (158)	MADRS ≥26CGI‐S ≥4MDE >3 months recurrent	Boulenger et al. (2014)
NCT01153009	8 weeks	VOR 15 (147)VOR 20 (154)DUL 60 (150)PBO (159)	MADRS ≥26CGI‐S ≥4MDE ≥3 months recurrent	Mahableshwarkar, Jacobsen, Chen, et al. (2015)
NCT01163266	8 weeks	VOR 10 (155)VOR 20 (150)PBO (157)	MADRS ≥26CGI‐S ≥4MDE ≥3 months recurrent	Jacobsen et al. (2015)
NCT01255787	8 weeks	VOR 5 (144)VOR 10 (148)VOR 20 (150)PBO (152)	MADRS ≥26CGI‐S ≥4MDE ≥3 months	Takeda (2013)
NCT00672958	6 weeks	VOR 5 (299)PBO (298)	MADRS ≥30MDE ≥3 months	Jain et al. (2013)
NCT00672620	8 weeks	VOR 2.5 (149)VOR 5 (153)DUL 60 (150)PBO (151)	MADRS ≥22MDE ≥3 months	Mahableshwarkar et al. (2013)
NCT01179516	8 weeks	VOR 10 (154)VOR 15 (151)PBO (160)	MADRS ≥26CGI‐S ≥4MDE >3 months recurrent	Mahableshwarkar, Jacobsen, Serenko, et al. (2015)

APTS, all patients treated set (*n* represents all randomized participants who took at least one dose of study medication); CGI‐S, Clinical Global Impression–Severity of Illness; DUL, duloxetine; MADRS, Montgomery‐Åsberg Depression Rating Scale; MDD, major depressive disorder; MDE, major depressive episode; PBO, placebo; VEN, venlafaxine; VOR, vortioxetine.

### 2.2 Scales and assessments

In each of the included studies, functional impairment was assessed using the SDS, a patient‐rated measure of functional disability that quantifies impairment with regard to (1) work/school activities, (2) social life and leisure activities, and (3) family relationships/home responsibilities using a discretized visual analog rating scale ranging from 0 (no impairment) to 1–3 (mild disability), 4–6 (moderate disability), 7–9 (marked disability), and 10 (extreme disability). The three item scores were added to produce the total score, which ranges from 0 to 30 (Sheehan & Sheehan, 2008; Sheehan et al., 1996). Functional remission was defined as an SDS total score ≤6, which was proposed by Sheehan and Sheehan (2008) and further supported by collateral analyses in large datasets by Sheehan and Sheehan (2008), Sheehan et al. (2008) and Sheehan et al. (2011) and which has been used in other studies of MDD to define functional remission (Mancini et al., 2012; Montgomery, Mansuy, et al., 2014; Sambunaris, Bose, et al., 2014; Sambunaris, Gommoll, et al., 2014; Soares et al., 2014).

### 2.3 Statistical analysis

A random‐effects, aggregated, study‐level meta‐analysis was performed using data for all therapeutic doses of vortioxetine (5, 10, 15, and 20 mg/day), with all clinical results reported as the least squares mean difference from placebo in the change from baseline to study endpoint for SDS total score, as well as for the three individual items. Analyses were conducted using the mixed‐effect model for repeated measures (MMRM) on a modified intent‐to‐treat population, the full analysis set (FAS; all randomized patients who received ≥1 dose of study medication and had ≥1 valid postbaseline value for the primary endpoint). The MMRM model utilized an unstructured covariance matrix and included terms for site, baseline SDS score by visit interaction, and treatment by visit interaction. ETRANK was performed as a sensitivity analysis on all randomized patients (true intent‐to‐treat population). ETRANK utilizes a nonparametric technique to incorporate timing and reasons for withdrawal into the analysis of incomplete repeated measures data (Entsuah, 1996). To account for heterogeneity in the results between studies, the random‐effects approach was chosen, conservatively broadening the confidence intervals (CIs) for the meta‐analysis results.

The level of heterogeneity was expressed in terms of *I* ^2^ (Higgins, Thompson, Deeks, & Altman, 2003), which describes the percentage of total variation in the treatment effect across studies that is due to heterogeneity rather than to chance. Standardized effect sizes (SES) were calculated as standardized mean differences (similar in interpretation to Cohen's *d*) based on analysis of covariance and MMRM results. Functional remission was evaluated using the last observation carried forward (LOCF) technique for imputing missing data. Patients with baseline SDS total score ≤6 (placebo: *n* = 168, vortioxetine: *n* = 308) were excluded from the remission analysis, since they were already in remission prior to beginning treatment. Odds ratios (ORs) were calculated using a two‐by‐two frequency table with 95% CIs. The numbers needed to treat (NNTs) were calculated as the reciprocals of the risk differences between vortioxetine and placebo.

To account for previously identified issues related to the SDS work score (Arbuckle et al., 2009; Coles et al., 2014; i.e, missing patient values due to unemployment during the study), sensitivity analyses were conducted in which scores on this individual item were calculated using three methods: (A) worst case I (“Case Report Form [CRF] Worst Case Score”, where work‐related information within the CRF is used to impute a missing work score); (B) imputed average non‐missing score (“Imputed Average Score”, where the average of the two available items—social and family—is used to impute the missing work item); and (C) worst case II (“Assumed Worst Case Score”, where a worst case score of 10 is assumed and imputed for all missing work items). Method A did not provide any additional information since no imputations were made with this rule in the entire database; therefore, it was identical to the nonimputation method used for the primary meta‐analysis. Missing data were imputed at baseline and all subsequent time points.

Additional post hoc analyses included the assessment of functional impairment in patients with severe MDD (MADRS total score ≥30 at baseline), patients with significant functional impairment (SDS total score ≥18 at baseline), both severe MDD and significant functional impairment (MADRS total score ≥30 and SDS total score ≥18 at baseline), and high levels of anxious symptoms (Hamilton Anxiety Rating Scale [HAM‐A; Hamilton, 1959] total score ≥20 at baseline). An exploratory analysis was conducted to evaluate the achievement of double remission in patients working at study endpoint (based on the FAS using observed cases). Double remission was defined as both symptomatic remission (MADRS total score ≤10) and functional remission (SDS total score ≤6) at study endpoint, and only included working patients (as defined by completion of the SDS work item) with baseline SDS total score >6 to avoid floor effects.

## 3 Results

A total of 4,216 patients (mean age 44 years) were randomized and received at least one dose of placebo (*n *=* *1,522) or vortioxetine 5–20 mg/day (*n *=* *2,694) in the nine clinical trials. Most patients (72.5%) had severe MDD (MADRS total score ≥30) at baseline. Demographic and baseline characteristics were similar in the placebo and vortioxetine treatment groups (Table 2), with women comprising approximately two‐thirds of the study population (65.8%).

**Table 2:** Summary demographics and baseline characteristics for patients included in the meta‐analysis (APTS)

	Placebo*N *=* *1,522	Vortioxetine*N *=* *2,694
Age (years), mean (*SD*)	43.92 (12.58)	44.43 (12.76)
Sex, *n* (%)		
Male	544 (35.7)	897 (33.3)
Female	978 (64.3)	1,797 (66.7)
Number of previous MDEs, mean (*SD*)	2.46 (2.32)	2.45 (2.54)
MADRS total score		
Mean (*SD*)	32.05 (4.08)	32.22 (4.11)
<30, *n* (%)	424 (27.9)	737 (27.4)
≥30, *n* (%)	1,098 (72.1)	1,957 (72.6)
HAM‐A total score, mean (*SD*)	19.45 (6.27)	19.73 (6.28)
CGI‐S score, mean (*SD*)	4.69 (0.67)	4.69 (0.65)
SDS total score	*n = *1,181	*n = *2,009
Mean (*SD*)	19.29 (6.04)	19.44 (5.97)
SDS work/school item score	*n = *1,181	*n = *2,010
Mean (*SD*)	6.05 (2.54)	6.26 (2.41)
SDS social item score	*n = *1,521	*n = *2,689
Mean (*SD*)	6.80 (2.21)	6.74 (2.20)
SDS family item score	*n = *1,521	*n = *2,690
Mean (*SD*)	6.62 (2.20)	6.57 (2.20)

APTS, all patients treated set (*n* represents all randomized participants who took at least one dose of study medication); CGI‐S, Clinical Global Impressions—Severity of Illness; HAM‐A, Hamilton Anxiety Rating Scale; MADRS, Montgomery‐Åsberg Depression Rating Scale; MDE, major depressive episode; *SD*, standard deviation; SDS, Sheehan Disability Scale.

### 3.1 SDS total score

**Figure 1:**
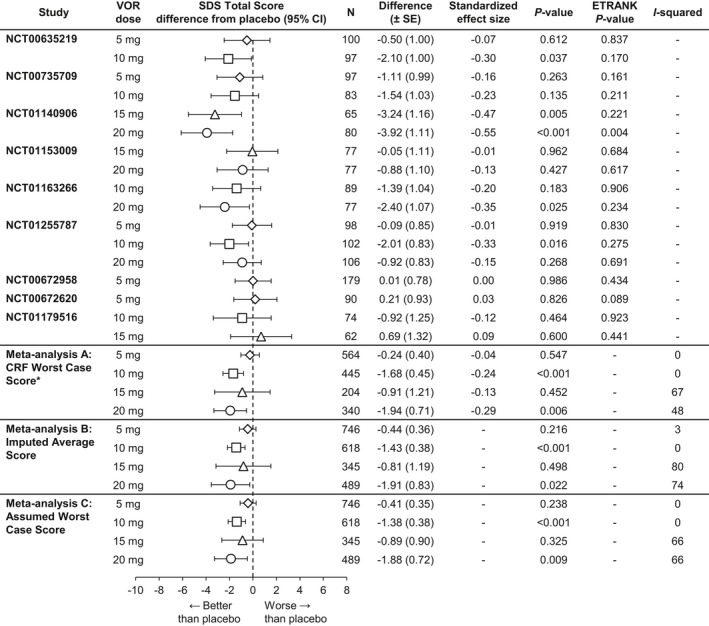
Meta‐analysis of the difference from placebo in Sheehan Disability Scale (SDS) total score change from baseline at week 6/8: Total population (full analysis set, mixed‐effect model for repeated measure). CRF, case report form; VOR, vortioxetine.*Primary meta‐analysis

The meta‐analysis of the change from baseline versus placebo on the SDS total score (FAS, MMRM) at study endpoint is summarized in Figure 1. Both vortioxetine 10 mg (*n *=* *445) and 20 mg (*n *=* *340) demonstrated statistically significantly greater improvements in patient functioning (SDS total score) relative to placebo (Δ −1.68, *p < *.001 and Δ −1.94, *p *=* *.006, respectively). Neither vortioxetine 5 mg (*n *=* *564, Δ −0.24, *p *=* *.547) or 15 mg (*n *=* *204, Δ −0.91, *p *=* *.452) separated from placebo. The overall SES supported the clinical relevance of the results of the change from baseline in SDS total score: −0.04 (5 mg), −0.24 (10 mg), −0.13 (15 mg), and −0.29 (20 mg). ETRANK analysis of the SDS total score after 6/8 weeks of treatment validated the findings of the MMRM analysis. The two alternative methods of imputation and the standard methodology (meta‐analysis A) produced similar estimates of treatment effect and identical conclusions.

### 3.2 SDS single‐item scores

**Figure 2:**
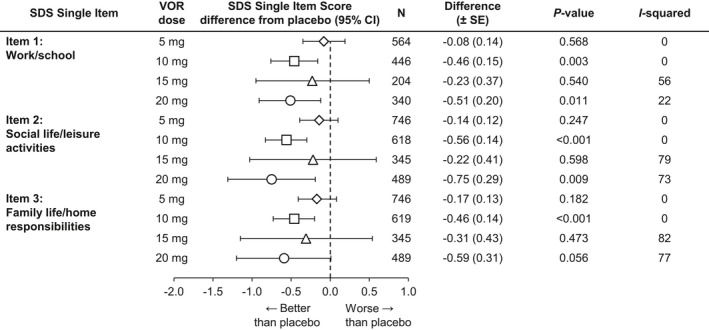
Meta‐analysis of the difference from placebo in Sheehan Disability Scale (SDS) single‐item scores change from baseline at week 6/8: Total population (full analysis set, mixed‐effect model for repeated measure). VOR, vortioxetine

The meta‐analysis of the difference from placebo in SDS single‐item scores at study endpoint is summarized in Figure 2. Vortioxetine 10 mg demonstrated statistically significantly greater improvements versus placebo for all three single items (work/school [*n = *446, Δ −0.46, *p *=* *.003], social life/leisure activities [*n *=* *618, Δ −0.56, *p *<* *.001], and family life/home responsibilities [*n = *619, Δ −0.46, *p *<* *.001]). Vortioxetine 20 mg demonstrated statistically significant differences from placebo for Item 1 (work/school [*n *=* *340, Δ −0.51, *p = *.011]) and Item 2 (social life/leisure activities [*n *=* *489, Δ−0.75, *p = *.009]). For Item 3 (family life/home responsibilities [*n = *489]), the difference was −0.59 (*p = *.056). The differences between vortioxetine 5 and 15 mg and placebo were not statistically significant.

### 3.3 Subgroup analyses

**Figure 3:**
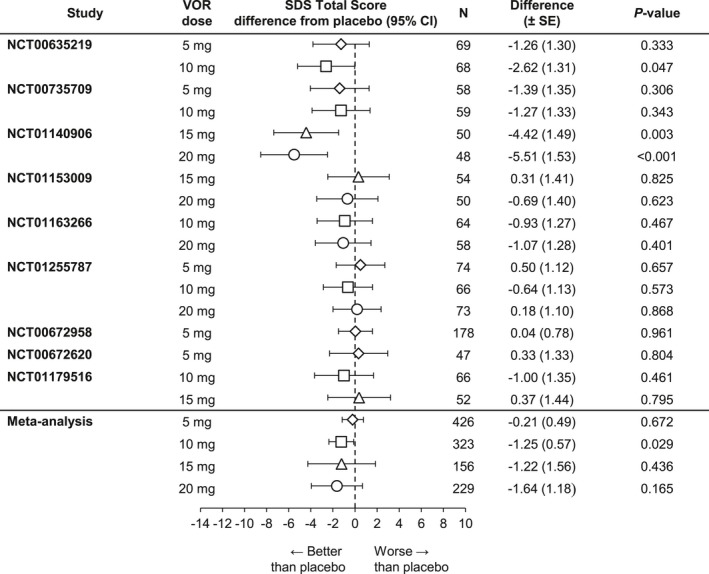
Meta‐analysis of the difference from placebo in Sheehan Disability Scale (SDS) total score change from baseline at week 6/8: Patients with baseline Montgomery‐Åsberg Depression Rating Scale (MADRS) ≥30 (full analysis set, mixed‐effect model for repeated measure). VOR, vortioxetine

**Figure 4:**
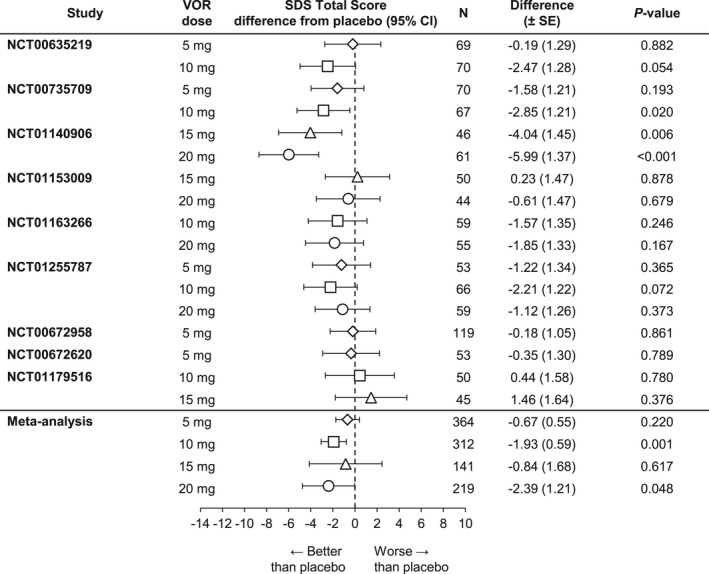
Meta‐analysis of the difference from placebo in Sheehan Disability Scale (SDS) total score change from baseline at week 6/8: Patients with baseline SDS ≥18 (full analysis set, mixed‐effect model for repeated measure). VOR, vortioxetine

**Figure 5:**
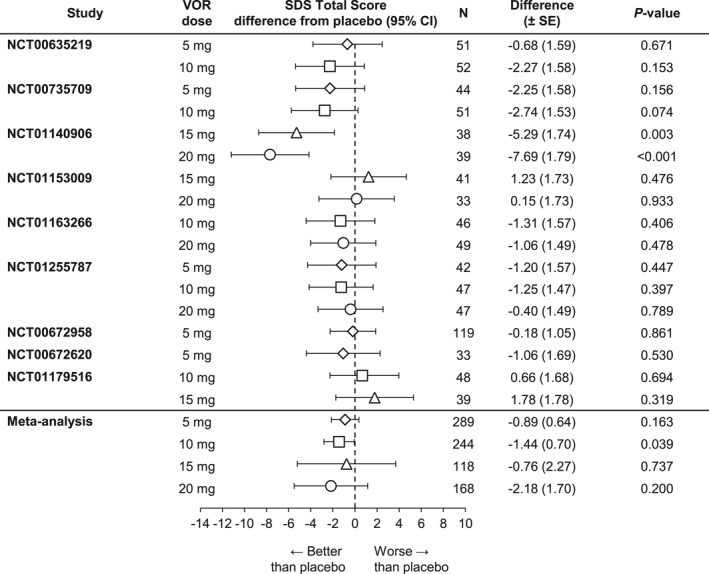
Meta‐analysis of the difference from placebo in Sheehan Disability Scale (SDS) total score change from baseline at week 6/8: Patients with baseline SDS ≥18 and baseline Montgomery‐Åsberg Depression Rating Scale (MADRS) ≥30 (full analysis set, mixed‐effect model for repeated measure). VOR, vortioxetine

**Figure 6:**
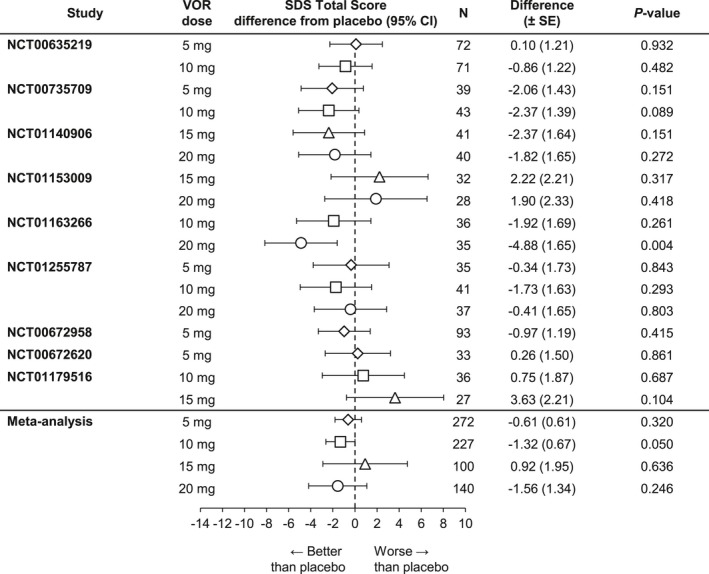
Meta‐analysis of the difference from placebo in Sheehan Disability Scale (SDS) total score change from baseline at week 6/8: Patients with baseline Hamilton Anxiety Rating Scale (HAM‐A) ≥20 (full analysis set, mixed‐effect model for repeated measure). VOR, vortioxetine

Results from the post hoc meta‐analysis indicated that the beneficial effects of vortioxetine on patient functionality extended to patients with severe depressive symptoms (MADRS total score ≥30) and/or significant functional disability (SDS total score ≥18) at baseline, as well as to those patients with significant level of anxiety symptoms at baseline (HAM‐A total score ≥20). Notably, vortioxetine 10 mg demonstrated statistically significant improvement on the SDS total score relative to placebo in all four subgroups (severe MDD symptoms, *n *=* *323, Δ −1.25, *p *=* *.029 [Figure 3]; significant functional impairment, *n *=* *312, Δ −1.93, *p *=* *.001 [Figure 4]; severe MDD and significant functional impairment, *n *=* *244, Δ −1.44, *p *=* *.039 [Figure 5]; and high level of anxiety symptoms, *n *=* *227, Δ −1.32, *p *=* *.050 [Figure 6]). A statistically significant difference from placebo was also found with vortioxetine 20 mg in the subgroup of patients with significant functional impairment at baseline (*n *=* *219, Δ −2.39, *p = *.048 [Figure 4]). The differences between vortioxetine 5 or 15 mg and placebo did not reach statistical significance in any subgroup analysis of the SDS.

### 3.4 Functional remission

**Figure 7:**
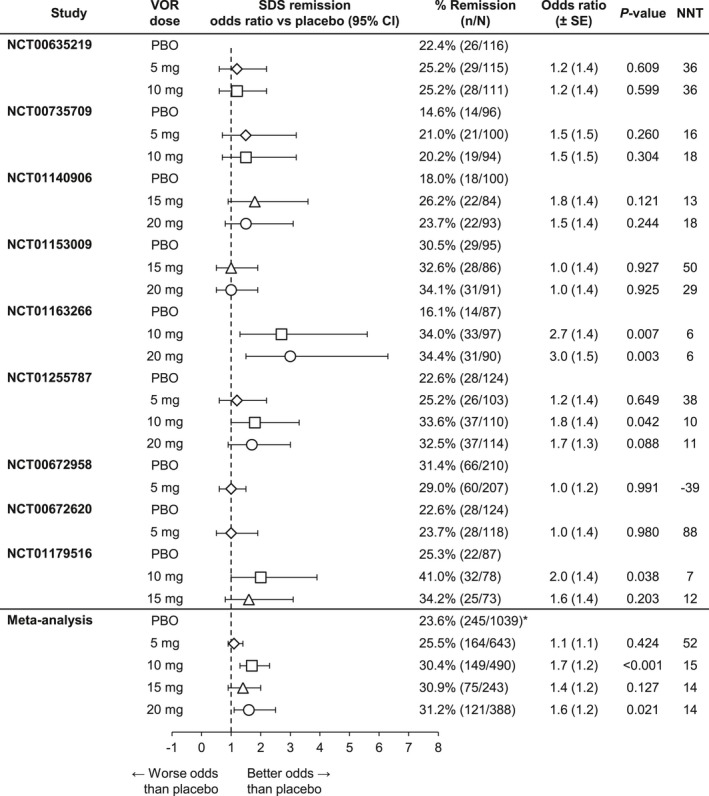
Meta‐analysis of remission rates (Sheehan Disability Scale [SDS] ≤6) at week 6/8: Patients with baseline SDS >6 (full analysis set, last observation carried forward). VOR, vortioxetine. *Overall rate for meta‐analysis placebo population

The meta‐analysis of functional remission (SDS total score ≤6) after up to 8 weeks of treatment in patients with baseline SDS total score >6 is summarized in Figure 7. Relative to placebo, the odds of achieving remission were statistically significantly greater with vortioxetine 10 mg (*n *=* *490, OR 1.7, *p *<* *.001) and 20 mg (*n *=* *388, OR 1.6, *p = *.021). There was no separation from placebo with vortioxetine 5 mg (*n *=* *643, OR 1.1, *p = *.424) or 15 mg (*n *=* *243, OR 1.4, *p = *.127). There was a slight dose–response trend in the analysis of NNT to achieve functional remission (vortioxetine 5 mg, NNT = 52; 10 mg, NNT = 15; 15 mg, NNT = 14; and 20 mg, NNT = 14).

### 3.5 Double remission

The meta‐analysis of double remission (MADRS total score ≤10 and SDS total score ≤6) in patients with baseline SDS total score >6 demonstrated that, relative to placebo (*n *=* *182, 17.5%), the odds of achieving composite remission were statistically significantly greater with vortioxetine 10 mg (*n *=* *112, 22.9%, OR 1.8, *p *<* *.001) and 20 mg (*n *=* *85, 21.6%, OR 1.6, *p *=* *.020), with nonsignificant benefits for vortioxetine 5 mg (*n *=* *119, 18.5%, OR 1.0, *p *=* *.764) and 15 mg (*n *=* *55, 22.7%, OR 1.3, *p *=* *.262). The benefits for vortioxetine that were observed in the meta‐analysis on double remission were supported by the results seen in the individual studies (data not shown).

## 4 Discussion

Major depressive disorder is associated with significant functional impairment in many areas, including social, occupational, and physical functioning. The effectiveness of MDD treatment in relation to functional outcomes is potentially confounded by the bidirectional relationship of depressive symptoms and functional impairment, as well as by premorbid functioning, which may not be accounted for when assessing the reduction in functional impairment (McKnight & Kashdan, 2009). The National Comorbidity Survey Replication found that 96.9% of patients with MDD for at least 12 months also suffered from functional impairment (in at least one area) associated with their depression, with 19.1% of patients reporting their impairment as “very severe” (Kessler et al., 2003). These respondents also reported being unable to work or fulfill daily activities for an average of 35.2 days in the past year of their MDD (Kessler et al., 2003). Understanding the relationship between MDD and functionality is important, especially considering the cost of lost work productivity due to absenteeism and presenteeism. In a study by Sheehan et al. (2011), patients with MDD experienced symptomatic remission more frequently than functional remission (38% and 32%, respectively; Sheehan et al., 2011), showing that depressive and functional symptoms do not always move in tandem and underscoring the need for physicians to address both types of symptoms. A recently published meta‐analysis of vortioxetine demonstrated significant improvement in depressive symptoms (as measured by the MADRS), with 46%–62% of patients responding to treatment across the doses (5–20 mg) and 28%–39% achieving remission (5–20 mg; Thase et al., 2016). In the current analysis, functional remission (SDS total score ≤6) at week 6/8 was 25.5%, 30.4%, 30.9%, and 31.2% for vortioxetine 5, 10, 15, and 20 mg, respectively. Symptomatic remission rates (MADRS total score ≤10) at these doses in the individual studies ranged from 28.8% to 36.0% for vortioxetine 5 mg, 21.4%–36.0% for vortioxetine 10 mg, 23.9%–34.9% for vortioxetine 15 mg, and 29.3%–38.4% for vortioxetine 20 mg, respectively (Baldwin et al., 2012; Boulenger et al., 2014; Henigsberg et al., 2012; Jacobsen et al., 2015; Jain et al., 2013; Mahableshwarkar, Jacobsen, Chen, et al., 2015; Mahableshwarkar, Jacobsen, Serenko, et al., 2015; Mahableshwarkar et al., 2013).

The inclusion of functional outcomes in clinical trials of antidepressant therapy is increasingly common. The SDS is a validated measure of functional impairment that has demonstrated sensitivity to impairment and the effects of treatment across a wide range of disorders, including MDD (Sheehan & Sheehan, 2008). The SDS has been used to assess functional improvement in association with improvements in depressive symptoms for duloxetine (Mancini et al., 2012; Oakes et al., 2012; Sheehan et al., 2011; Wise et al., 2008), desvenlafaxine (Dunlop et al., 2011; Guico‐Pabia et al., 2012; Soares et al., 2009), paroxetine (Wise et al., 2008), bupropion (Hewett et al., 2010; Soczynska et al., 2014), escitalopram (Romera et al., 2012; Soczynska et al., 2014), venlafaxine (Fann et al., 2015; Hewett et al., 2010), levomilnacipran (Asnis et al., 2013; Sambunaris, Bose, et al., 2014), and agomelatine (Montgomery, Nielsen, et al., 2014; Zajecka et al., 2010) with results that have been variable with respect to clinical significance; however, many of these antidepressants showed significant differences versus placebo in the change from baseline versus placebo in the SDS total score when patients were stratified by baseline depressive symptom severity. A recent pooled analysis showed that treatment with duloxetine (*n* = 1,029) and SSRIs (*n* = 835) resulted in significantly greater improvements in the SDS total score (∆ −1.9, *p *<* *.001; ∆ −1.7, *p *<* *.01, respectively) compared to placebo (*n* = 329). Further, higher SDS and higher Hamilton Depression Rating Scale (17‐item) baseline scores predicted a lower probability of functional improvement after active treatment (*p *<* *.0001; *p *<* *.01, respectively; Sheehan et al., 2016). In general, treatment with vortioxetine has demonstrated a similar quantitative effect in patients with MDD. In a direct comparison to agomelatine (25–50 mg) in patients with an inadequate response to SSRI/SNRI monotherapy, vortioxetine (10–20 mg) had significantly greater reductions on the SDS total and item scores after 8 and 12 weeks of treatment (Montgomery, Nielsen, et al., 2014). Vortioxetine 10 mg has also demonstrated numerically larger changes from baseline on the SDS total and item scores than venlafaxine XR 150 mg/day after 8 weeks of treatment in adult patients with MDD in Asia (Wang, Gislum, Filippov, & Montgomery, 2015).

This meta‐analysis of SDS data from the vortioxetine clinical trial program indicates that vortioxetine 10 and 20 mg daily provided statistically significant and clinically relevant improvement in patient functioning in short‐term studies, as measured by (1) the change in SDS total score from baseline to study endpoint versus placebo and (2) the achievement of functional remission (SDS total score ≤6). Additionally, vortioxetine 10 mg daily demonstrated clinically significant benefits on all aspects of functioning (SDS single‐item scores) in the total population as well as on overall functioning (SDS total score) in patients with severe MDD and/or significant functional impairment or high anxiety symptoms at baseline. These findings are consistent with the results of the individual studies, where vortioxetine administration was associated with improvements in patient functioning as well as in depressive symptoms (Baldwin et al., 2012; Boulenger et al., 2014; Henigsberg et al., 2012; Jacobsen et al., 2015; Jain et al., 2013; Mahableshwarkar, Jacobsen, Chen, et al., 2015; Mahableshwarkar, Jacobsen, Serenko, et al., 2015; Mahableshwarkar et al., 2013; Takeda, 2013).

Functional improvement with vortioxetine 5–20 mg in the current analysis tended to be dose‐dependent, with the exception of the 15‐mg dose. No statistically significant improvements in function were demonstrated for the vortioxetine 5‐ or 15‐mg treatment groups. Further investigation is warranted to explain these findings; however, it is notable that vortioxetine 15 mg was evaluated in only three of the nine studies included in the present meta‐analysis (Boulenger et al., 2014; Mahableshwarkar, Jacobsen, Chen, et al., 2015; Mahableshwarkar, Jacobsen, Serenko, et al., 2015), and a statistically significant difference from placebo in the change in depressive symptoms and functionality was demonstrated in only one of the three individual studies (Boulenger et al., 2014). Heterogeneity between studies was also greater at the 15‐mg dose level (*I* ^2^ = 67%; *p *=* *.47) than at any other dose level (0%, 0%, and 48% for 5, 10, and 20 mg, respectively).

This analysis employed three alternative methods of imputation to account for missing work scores due to unemployment during the study. All three methods produced similar estimates of treatment effect on SDS total score, providing additional insights into how differing statistical methodology can be utilized to address the issues raised regarding missing work scores.

Results of the current analysis suggest that improvement in overall functioning with vortioxetine is also observed for those patients with severe MDD and/or significant functional impairment and those with high anxiety symptoms at baseline. Previous antidepressant studies conducted in MDD patients with high anxiety symptoms suggest that these patients are often difficult to treat, exhibiting a slower or less robust response, with a higher risk of adverse events and suicidal ideation (Andreescu et al., 2007; Fava et al., 2008).

The results of this analysis add to the growing body of evidence that suggests that, in addition to improving depressive symptoms, vortioxetine provides functional benefits in patients with MDD. Pooling of data from multiple, smaller—but relatively similarly designed—clinical trials resulted in a large sample size and greater statistic power to explore these effects. There are, however, limitations to this analysis that should be considered when interpreting these results. First, this was an analysis performed on data from studies that were not powered to show statistical differences in SDS between vortioxetine and placebo. Second, individual studies used different doses and utilized different primary efficacy endpoints. Last, all data were from short‐term vortioxetine clinical studies. In light of the demonstrated asynchrony between depressive symptoms and patient functioning, the prudence of assessing function after such a short course of treatment is debatable.

## 5 Conclusion

In this meta‐analysis of data from nine short‐term (6/8‐week) clinical studies conducted in adults with MDD, treatment with vortioxetine 10 or 20 mg daily was associated with greater improvement in patient functioning and a greater likelihood of achieving functional remission compared to placebo.

## Conflict of Interest

Ioana Florea, Henrik Loft, Natalya Danchenko, Benoit Rive, and Melanie Brignone are employees of H. Lundbeck A/S. Elizabeth Merikle and Paula L. Jacobsen are employees of Takeda Pharmaceuticals Company, Ltd. David V. Sheehan reports receiving grants from the University of South Florida College of Medicine during the conduct of the study and is a copyright holder of the Sheehan Disability Scale used in this study. [Correction added on 16 February 2017, after first online publication: the Conflict of Interest statement has been inserted.]
